# The discovery of novel variants reveals the genetic diversity and potential origin of Seoul orthohantavirus

**DOI:** 10.1371/journal.pntd.0012478

**Published:** 2024-09-12

**Authors:** Guopeng Kuang, Jing Wang, Yun Feng, Weichen Wu, Xi Han, Genyang Xin, Weihong Yang, Hong Pan, Lifen Yang, Juan Wang, Mang Shi, Zihou Gao

**Affiliations:** 1 Yunnan Provincial Key Laboratory for Zoonosis Control and Prevention, Yunnan Institute of Endemic Disease Control and Prevention, Dali, Yunnan, China; 2 The Centre for Infection and Immunity Study, School of Medicine, Shenzhen Campus of Sun Yat-sen University, Shenzhen, Guangdong, China; 3 College of Global Change and Earth System Science, Beijing Normal University, Beijing, China; 4 School of Public Health, Dali University, Dali, Yunnan, China; The University of Hong Kong, CHINA

## Abstract

Seoul orthohantavirus (SEOV) has been identified as one of the main causative agents of hemorrhagic fever with renal syndrome (HFRS) in China. The virus was found circulating in rodent populations in almost all provinces of the country, reflecting the wide distribution of HFRS. Here, using the direct immunofluorescence assay (DFA) and real-time quantitative reverse transcription PCR (qRT-PCR) approach, we performed screening in 1784 small mammals belonging to 14 species of three orders captured in the main areas of HFRS endemicity in Yunnan province (southwestern China) and identified 37 SEOV-positive rats (36 *Rattus norvegicus* and 1 *Rattus tanezumi*). A 3-year surveillance of HFRS epidemics and dynamics of rodent reservoir density and virus prevalence implied a potential correlation between them. The subsequent meta-transcriptomic sequencing and phylogenetic analyses revealed three SEOV variants, among which two are completely novel. The ancestral character state reconstruction (ACSR) analysis based on both novel variants and documented strains from 5 continents demonstrated that SEOV appeared to originate near the southwestern area (Yunnan-Kweichow Plateau) of China, then could spread to other regions and countries by their rodent carriers, resulting in a global distribution today. In summary, these data furthered the understanding regards genetic diversity and the potential origin for SEOV. However, the expanding endemic foci in the province suggest that the virus is spreading over a wider region and is much more diverse than previous depicted, which means that increased sampling is necessary.

## Author summary

Hantaviruses are primarily hosted by small mammals such as rodents, shrews, moles, and bats. In recent years, the diseases caused by rodent-associated hantaviruses become more frequent among humans in Yunnan province. In order to curb human infections, it is necessary to monitor the dynamics of virus prevalence in their natural reservoir populations. As expected, we detected 37 SEOV strains harbored by rats in the main endemic areas of the province, and the subsequent meta-transcriptomic and phylogenetic analyses revealed two SEOV variants that diverged early from the rest of the viruses, based on which Yunnan-Kweichow Plateau appears to be the region where earlier SEOV was originated. Further studies and more samples are needed to clarify how the virus migrates worldwide, and the pathogenicity of newly discovered viruses.

## Introduction

Hantaviruses are enveloped RNA viruses belonging to the family *Hantaviridae* and order *Bunyavirales*, containing a negative sense tri-segmented genome, namely large (L), medium (M), and small (S) segments [1]. The latest taxonomical proposal lists 53 species to the family, which are primarily hosted by a broad range of mammalian species of the orders *Rodentia*, *Eulipotyphla*, and *Chiroptera* [2–4], although piscine and reptilian hosts also recognized for more divergent members of the family [5,6]. Within genus *Orthohantavirus*, the ecology and geographic distribution of a few members resembles those of their natural host reservoir, namely rodents, resulting in a wide distribution across different continents [7,8].

Some of the rodent-borne hantaviruses have been identified as the etiological agent(s) of hemorrhagic fever with renal syndrome (HFRS) and hantavirus pulmonary syndrome (HPS) in humans [1,7]. To date, only Hantaan orthohantavirus (HTNV) and Seoul orthohantavirus (SEOV) have been found to cause HFRS in China [9–11] and estimated that approximately ten thousand of human infections each year recently [12]. Notably, SEOV has been found in almost all major HFRS areas of endemicity in China, emphasizing it as the most important hantavirus possessing threats to public health in the country [9–11,13]. Similarly, Yunnan province has been characterized by one of the natural foci of HFRS, resulting from the co-circulation of HTNV and SEOV [14–16]. Within the province, Xiangyun County and Chuxiong City emerged as the main areas of HFRS endemicity, recording 810 cases (44.16% of the total 1834 cases) and 303 cases (16.52% of the total 1834 cases), respectively, over the period from 2012 to 2020 [15]. Additionally, Luxi County was previously charactered as a natural focus, with 126 cases (14.38% of the total 876 cases) reported from 1976 to 2012 [16]. The members of the family *Hantaviridae*, including two rodent-borne hantaviruses (Luxi orthohantavirus and Fugong orthohantavirus) and one insectivore-borne hantavirus (Lianghe virus), were previously discovered in this province [17–19]; however, the pathogenicity of these viruses remains unclear.

Pathogenic hantaviruses, each linked to one or few closely related reservoir host species, are widely considered to have co-evolved with rodent hosts over millions of years [20,21]. However, it is no coincidence that frequent host-switches (cross-species transmission) among wildlife species in the long evolutionary history of the virus [19,22,23]. Similarly, the rapid increasing studies reveal SEOV is primarily hosted by *Rattus norvegicus*, occasionally spillover transmitting to the secondary hosts such us *Rattus tanezumi*, *Rattus rattus*, and *Rattus nitidus* [20,24–27]. Currently, there are at least seven phylogenetic lineages/subgroups of SEOV have been identified [28]; however, most of the variants are genetically homogeneous and closely related to each other, with no apparent geographical or host structure on the phylogeny [27]. In addition, cross-species genomic reassortment events between SEOV and HTNV were previously reported [29], which further complicated the current understanding of virus diversity.

The zoonotic epidemics for hantavirus involve multiple phases, including disease dynamics in the reservoir host, pathogen exposure, and the within-human factors that affect susceptibility to infections. However, undoubtedly, “pathogen pressure” is one of the most critical factors determining the spillover transmission to humans [30]. Human infections with hantaviruses usually result from inhalation of aerosolized rodent excreta containing these pathogens in a suitable environment for transmission [1,7]. Accordingly, current efforts to curb hantavirus infection are focusing on avoiding contact between humans and host rodents [31,32], and the use of vaccines [7]. There is high correlation between rodent population density and hantavirus prevalence within rodents, which in turn affect the risk of HFRS in humans [33–36]. Furthermore, environmental variability and climate changes have also been characterized as factors affecting hantavirus infections through impact on the rodent reservoir populations [35–40]. Therefore, monitoring the factors such as rodent abundance, virus infection intensity, and even environmental and climatic conditions is vital for evaluating the risk of HFRS.

HFRS is an essential and ongoing public health issue in China [41] and especially in the Southwestern Yunnan province [15,16,42–45]. However, due to the lack of sequencing data, it is still unclear the genetic diversity, geographic distribution, temporal dynamics and evolution of the causative viruses, namely, HTNV and SEOV. Therefore, we performed a surveillance in the major endemic areas for HFRS in Yunnan for three years, aiming to characterize disease dynamics in human populations, the prevalence and diversity of hantaviruses among reservoir animals, and the link between the two.

## Materials and methods

### Ethics statement

The procedures and protocols of sample collection and processing in this study were reviewed and approved by the Medical Ethics Committee of the Yunnan Institute of Endemic Diseases Control and Prevention (file 20230003). All experiments were performed with approval by the Biosafety Committee of the Yunnan Institute of Endemic Diseases Control and Prevention. All sample processing and nucleic acid extraction procedures followed the biosafety guidelines.

### Samples collection and classification

Small animals were captured with snap-traps or cage-traps in residential areas and field areas of the major HFRS regions (Chuxiong and Xiangyun) of endemicity and and Luxi County in Yunnan province from 2019 to 2021. Correspondingly, a total of 45, 37, and 22 trapping sites were set up in Chuxiong, Xiangyun, and Luxi, respectively. Animal species were identified by experienced field biologists on capture based on the morphological traits and the information regarded location, date, habitat, and the number of traps was also recorded. The alive captured animals were anesthetized with ether and the lung tissues were retrieved, then stored immediately in liquid nitrogen and transported back to the laboratory for the subsequent screening.

### Hantaviruses detection

Hantaviral antigens present in the lung tissue samples were initially screened by direct immunofluorescence assay (DFA) as previously described [10]. Briefly, the tissues were cut into 4-um sections and stained with rabbit anti-SEOV/Z37 or HTNV/Z10 antibodies labeled with fluorescein isothiocyanate (FITC), which was provided by Chinese Center for Disease Control and Prevention, then granular fluorescence observed in the cytoplasm was considered positive for hantaviruses [46]. Subsequently, total RNA was extracted and purified from the DFA-positive tissues using RNeasy Plus Universal Mini Kit (Qiagen, Germany). The initial screening was confirmed by real-time quantitative reverse transcription PCR (qRT-PCR) by using Detection Kit for HTNV and SEOV (PCR-Fluorescence Probing) (Shenzhen Aodong Inspection&Testing Technology Co., Ltd., China) and processed by LightCyclear 480 (Roche, Switzerland) following the instruction provided by the manufacturer.

The partial sequences of SEOV were also recovered by nested RT-PCR, using a set of degenerate primers as described previously—HV-SFO (GGCCAGACAGCAGATTGG) and HV-SRO (AGCTCAGGATCCATGTCATC) for the 1st-round reactions, while SEO-SF (TGCCAAACGCCCAATCCA) and SEO-SR (GCCATCCCTCCGACAAACAA) for the 2nd-round PCR [47]. All PCR products were purified and subsequently sequenced with both forward and reverse primers by Sangon Biotech. Sequences were assembled and compared using the SeqMan and MegAlign programs, respectively, which were implemented in the DNASTAR software package (Lasergene).

### Human HFRS data collection

Data on HFRS cases in Chuxiong, Xiangyun, and Luxi from 2019 to 2021 were obtained from the National Notifiable Disease Surveillance System (NNDSS), a passive surveillance system reported by local People’s Hospitals and Centers for Disease Control and Prevention (CDC). The information regarding sex, age, occupation, residential address, and onset date of symptoms for each case was recorded. All HFRS cases were first diagnosed based on clinical symptoms defined by a national standard, and the subsequent diagnosis was confirmed by detecting specific IgM and IgG antibodies against hantaviruses [15,16,42].

### Viral genome and rodent COI gene sequencing

Ten representative samples were selected to perform the meta-transcriptomics sequencing. Accordingly, a total of ten RNA libraries were constructed by using Zymo-Seq RiboFreem TM Total RNA library kit (Zymo Research, USA) following the instruction provided by the manufacturer. All RNA libraries were sequenced using the Illumina Novaseq 6000 platform. Low-quality reads for all libraries were removed first by using Trimmomatic [48] followed *de novo* assembly using the Trinity program [49]. The assembled contigs were then compared against the National Center for Biotechnology Information (NCBI) non-redundant protein (nr) database using DIAMOND BLASTx, with an e-value threshold of 1×10^−5^ [50]. The contigs relevant to genome segments of SEOV and COI gene of rodents were identified from all included RNA libraries. Rodent species identification was carried out by comparing the COI sequences against to the online BOLD database (http://www.boldsystems.org/index.php/IDS_IdentificationRequest). The very termini of each segment was determined by mapping against the reference sequences (NC_005236 to NC_005238, Seoul orthohantavirus strain 80–39) using Bowtie2 [51]. Abundance of viral genome and rodents COI gene was estimated number of reads mapped to confirmed sequences [51]. Potential open reading frames (ORFs) and coding arrangements for the obtained viral genomes were predicted using ORFfinder (https://www.ncbi.nlm.nih.gov/orffinder/) and confirmed by online blastp program (https://blast.ncbi.nlm.nih.gov/Blast.cgi).

### Phylogenetic analyses

To investigate the evolutionary relationships among both novel viruses and the known strains, the phylogenetic trees of newly detected viruses in this study were based on partial sequences only, and the representative strains compared with known strains based on complete ORFs were reconstructed. Published nucleotide sequences for all segments of the currently validated seven lineages of SEOV were downloaded from the NCBI website (S3 Table). MAFFT was used to align the nucleotide sequences [52], the terminal sequences were removed manually, and ambiguously aligned sequences were removed using trimAl [53]. Phylogenetic trees were reconstructed using maximum likelihood (ML) method implemented in PhyML program, with GTR+G substitution model and SPR tree topology optimization algorithm [54].

### Ancestral character state reconstruction

The MCMC tree, based on partial M segment sequences of SEOV from GenBank (S4 Table) and new sequences obtained in this study, was inferred by the program MrBayes version 3.2.7 using GTR model with gamma-distributed rate variation across sites and a proportion of invariable sites [55]. The analysis was evaluated for 1 million generations when the average standard deviation of split frequencies is below 0.01, sampled every 1000 iterations. The final tree was summarized sampled trees after the initial 25% were discarded as burn-in. Subsequently, Ancestral host and biogeographic distribution of each lineage were reconstructed based on the tree samples mentioned above using a maximum-likelihood approach in Mesquite 3.70 with a Markov k-state 1 parameter (Mk1) model [56, 57]. In this approach, an equal and symmetrical rate of change between any two states was assumed, and the character state frequencies were estimated from the transition probabilities and the uniquely best state was displayed.

## Results

### Collection of small mammals

**Fig 1:**
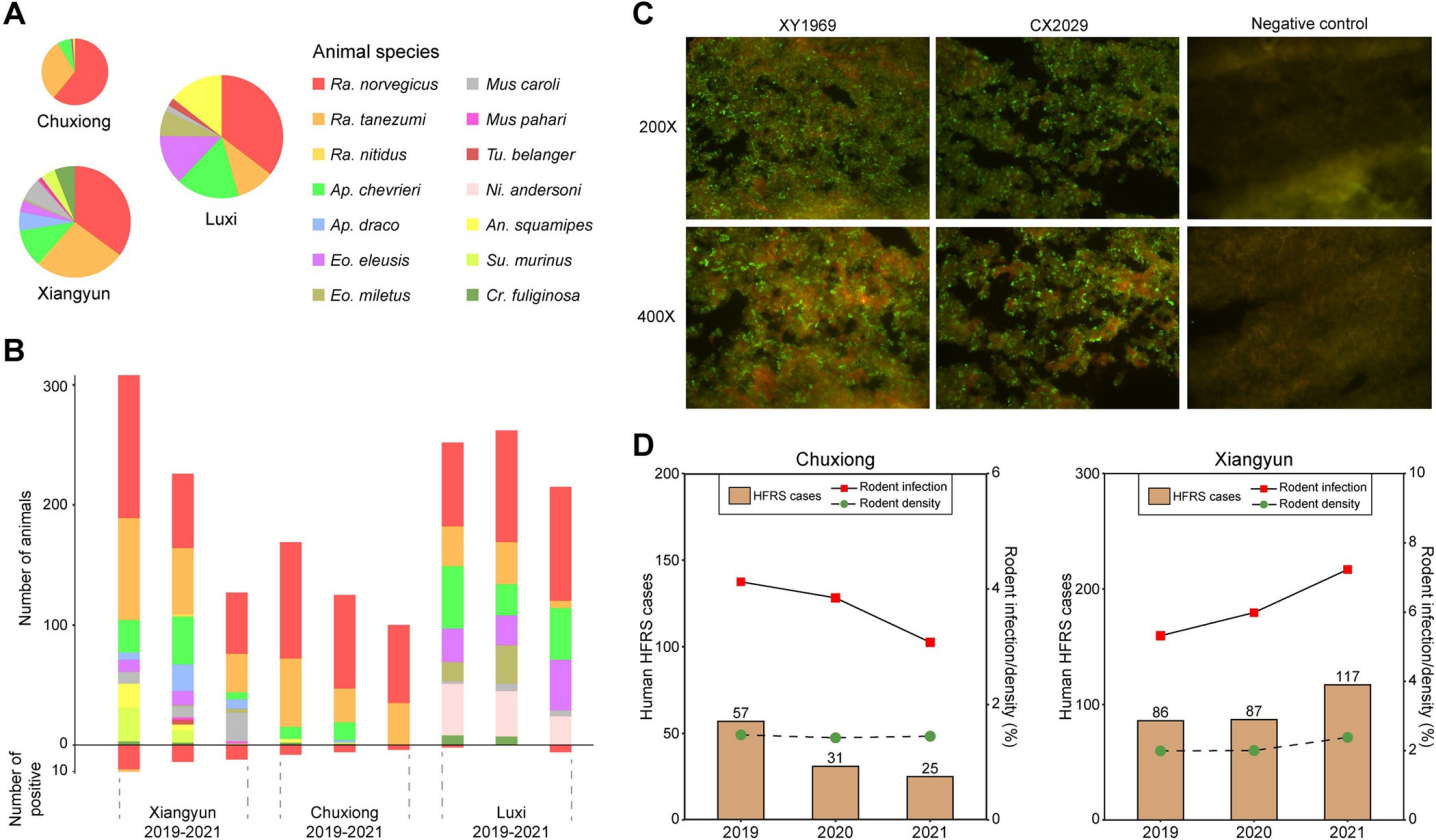
Overview of animal hosts, hantavirus detection, and human HFRS epidemics in this study. (A) Amounts and proportion of small mammals at the species level for three surveillance counties. (B) Animal composition of each surveillance county from 2019 to 2021. (C) Detection of Hantaviral antigen for samples XY1969 and CX2029 using a direct immunofluorescence assay (DFA). (D) Comparison of human HFRS cases, the densities and prevalence rates of hantavirus among rodent reservoirs in Chuxiong and Xiangyun from 2019 to 2021.

From 2019 to 2021, a total of 1784 small mammals (1588 rodents, 173 shrews and 23 tree shrews) were captured in both residential and field habitats of three counties (Chuxiong, Xiangyun, and Luxi), which are the main endemic areas for HFRS in Yunnan Province, China. Animal species information was identified by experienced field biologists on capture based on morphological criteria—14 species of small mammals belonging to three orders (*Rodentia*, *Eulipotyphla*, and *Scandentia*) were classified (S1 Table). Most of the collected animals are Norway rats (*Rattus norvegicus*, 40.92%) and Oriental house rats (*Rattus tanezumi*, 20.52%) (Fig 1A and 1B), which is also the predominant species of rodents in the monitoring sites.

### Detection of hantaviruses

Hantaviral antigens in the lung tissues of all 1784 trapped animals were individually tested by the direct immunofluorescence assay (DFA) using antibodies against the SEOV and HTNV. The granular fluorescence observed in the cytoplasm was considered existence of antigenic properties for hantaviruses (Fig 1C). Accordingly, a total of 42 samples were positive for viral antigen detection. Based on the initial screening, all antigen-positive tissues were cross-examined with quantitative RT-PCR (qRT-PCR) assay. As a result, 37 of 42 samples were positive for the SEOV (none of HTNV was detected), while the remaining five samples were negative. For the following analyses, only those positive for both DFA and qRT-PCR assay were considered positive. Therefore, the prevalence rates are 24/661 (3.63%), 9/394 (2.28%), and 4/729 (0.55%) for Xiangyun, Chuxiong, and Luxi, respectively (Table 1).

**Table 1:** Prevalence of hantaviruses among rodent reservoirs and human HFRS epidemics in the main endemic areas of Yunnan.

Location	Date	NO. of reservoir rodents	NO. of DFA-positive samples	NO. of PCR-positive samples	Prevalence rates of reservoir rodents	Human HFRS cases	Reservoir rodent density
Xiangyun	2019	204	12	11	5.39	86	1.99
	2020	117	7	7	5.98	87	2.02
	2021	83	6	6	7.23	117	2.31
Chuxiong	2019	97	5	4	4.12	57	1.47
	2020	78	4	3	3.85	31	1.42
	2021	65	3	2	3.08	25	1.45
Luxi	2019	70	1	1	1.43	0	-
	2020	93	1	0	0.00	0	-
	2021	97	3	3	3.09	0	-
**Total**		**904**	**42**	**37**	**4.09**	**403**	**-**

### Rodent population dynamics and human HFRS epidemics

All 37 hantavirus-positive rats were identified as two species: 23, 9, and 4 Norway rats from Xiangyun, Chuxiong, and Luxi, respectively, and only one Oriental house rat from Xiangyun (Fig 1B). Accordingly, prevalence rates of rodents in Xiangyun ranged from 5.39% to 7.23%, whereas the rates are lower (i.e. 3.08% to 4.12%) in Chuxiong (Table 1). During the study, the annual rodent reservoir densities (indicated by the capture rate of rodents per hundred trap-nights) of Xiangyun from 2019 to 2021 were 1.99%, 2.02%, and 2.31%, while those of Chuxiong were 1.47%, 1.42%, and 1.45% (Table 1). We failed to evaluate the rodent densities in Luxi because of incomplete information on traps.

The Human HFRS data were obtained from the National Notifiable Disease Surveillance System (NNDSS), which revealed the different trends for the two counties considered here: the prevalence of HFRS in Chuxiong appeared to decrease from 2019 to 2021, with disease cases of 57, 31, and 25 documented; whereas in Xiangyun, it displayed an upward trend in increasing numbers of 86, 87, and 117 cases (Table 1). Of note, the number of HFRS cases, the positive rates of SEOV among rodents as well as the rodent density showed very similar trend in Xiangyun (Fig 1D). However, in Chuxiong county, HFRS cases are only correlated with SEOV prevalence, but not with the rodent density. Despite these differences, strong correlations were observed between HFRS cases, rodent density, and SEOV prevalence in both counties, with Spearman’s ρ ranging from 0.94 to 1 (p < 0.05) (S1 Fig).

### Molecular epidemiological characteristics of the detected hantaviruses

**Fig 2:**
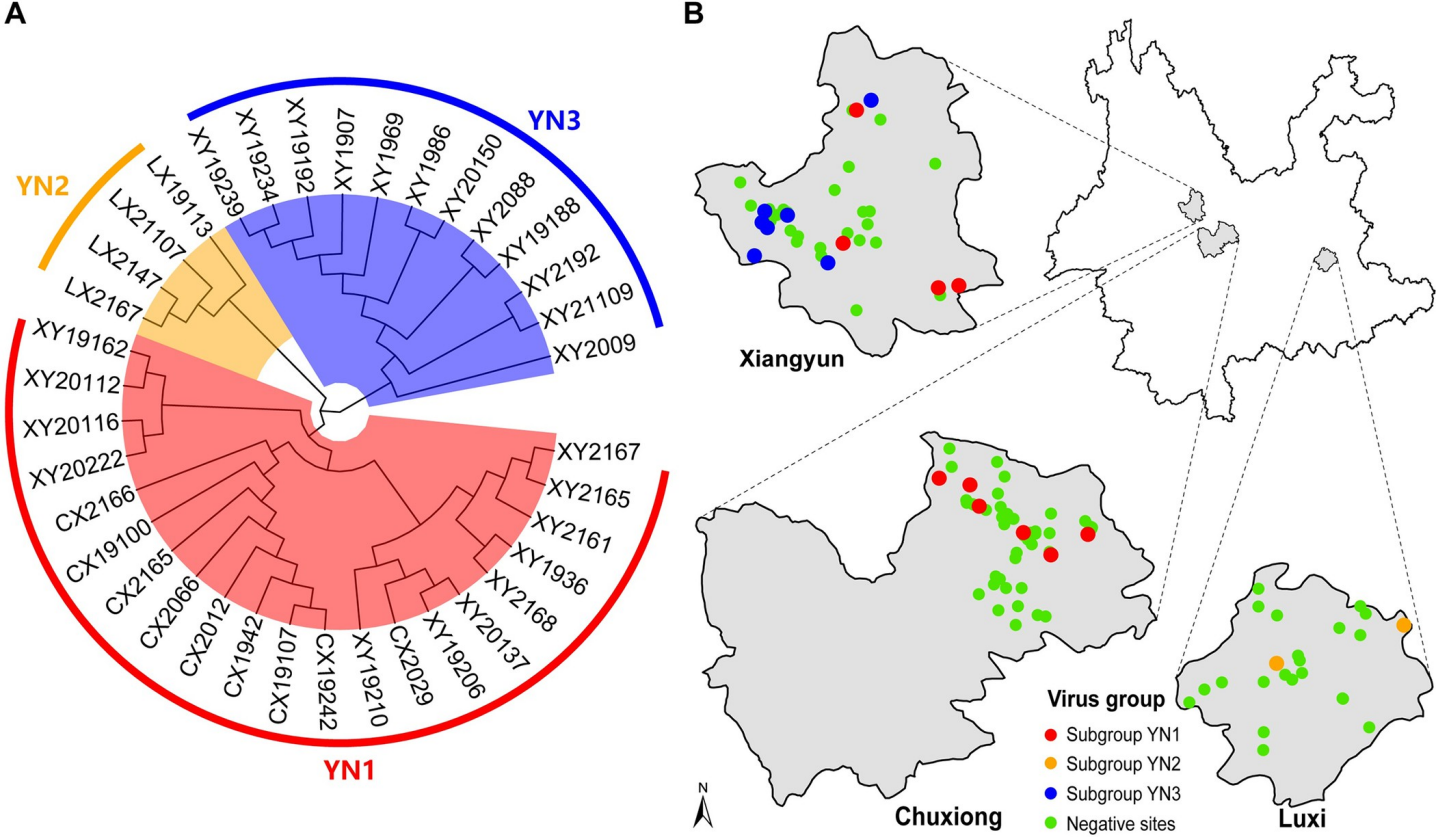
Molecular epidemical characteristics of Seoul orthohantavirus in three surveillance counties. (A) ML tree based on partial nucleotide sequences (approximately 437 bp) of S segment for all 37 SEOV strains detected in this study. Different colored blocks represent different virus groups: red (subgroup YN1), yellow (YN2), and blue (YN3). (B) Sampling sites, positive and negative animal geographical distributions in Xiangyun, Chuxiong, and Luxi County. The basemap shapefile used in ArcGIS was sourced from the publicly accessible GADM dataset (https://gadm.org/download_country.html). Sampling sites were represented by dots colored according to the virus-infection status.

All positive samples revealed by RT-PCR were subject to sequencing, which revealed approximately 437 bp of the S segment. Sequence similarity comparisons based on the limited length of generated sequences revealed close relationships among these viruses, which exhibited 97.1% to 100% amino acid and 84.2% to 99.7% nucleotide identities. The phylogenetic analyses of the newly obtained sequences revealed three distinct clusters (i.e. Subgroup YN1, YN2 and YN3) (Fig 2A). Notably, the viral sequence (XY19192) from Oriental house rats shared 99.7% nucleotide identity with those hosted by Norway rats (Fig 2A), implying inter-species transmission between two different rodent species. The subgroup YN2 variants (n = 4, 2 trapping sites) were only detected only in Luxi, subgroup YN3 variants (n = 12, 8 trapping sites) were only from Xiangyun, whereas subgroup YN1 variants were discovered in both Chuxiong (n = 9, 6 trapping sites) and Xiangyun (n = 12, 4 trapping sites) (Fig 2B). Subgroup YN1 and subgroup YN3 of SEOV were both detected from Xiangyun, suggesting the co-circulation of different viral variants in the same endemic areas. Besides, all trains of the two variants are hosted by the same rodent species, namely *Rattus norvegicus*. Almost all PCR SEOV-positive rats were captured in residential areas, and only one host (XY21109) is a wild rat.

### Characterization of the virus genome

In order to further characterize the genome of viruses, we performed meta-transcriptomic sequencing on ten positive samples (each from different trapping site), including seven samples from Xiangyun, two samples from Chuxiong, and one sample from Luxi. Overall, 25893504 (XY2009) to 85653198 (CX19242) clean reads were generated for each library (S2 Table). All clean reads were assembled *de novo*, and the obtained contigs were compared against NCBI non-redundant (nr) databases. According to the taxonomy annotation of all contigs, the full-length genome sequences of SEOV and rodent cytochrome c oxidase I (COI) gene from each library were recovered with 101.4 to 1161.4 folds coverage (S2 Table). The complete sequences of the COI gene were further analyzed by comparing against the BOLD database and demonstrated the same results as those of morphological identification (S2 Table). As for viruses, the full-length genomes (approximately 11956 nt) of SEOV were obtained from each library, with lengths of 1772 nt, 3654 nt, and 6530 nt for the separate segments of S, M, and L, respectively, and the viral RNA share a 3’ terminal sequence (3’-UAGUAGUAGACU) and 5’ terminal sequence (5’-AUCAUCAUCUGAG) for all three segments.

On the genomic scale, subgroup YN1, 2, and 3 shared 83.9%-87.4% nucleotide identity to each other, while the within subgroup diversity is small (> 99.0% nucleotide identity). We then compared these viruses with previous published sequences, YN3 shared close relationship with known viruses (GZRn54, 98.7%-98.8% identity), whereas YN1 and YN2 were distantly related to existing strains, with 82.8%-96.7% nucleotide identity and 96.1%-100% acid amino to reference strain 80–39 in South Korea. The closest strain to YN1 was Seoul virus L0199 (93.5% identity, partial nucleocapsid protein gene) sampled from Laos, whereas the closest strain to YN2 was Hantavirus CGRn8316 (89.3% identity, M segment of a SEOV-HTNV reassortant) sampled from Guizhou province, China. Unfortunately, both L0199 and CGRn8316 reference strains lacked the complete genome sequences.

### Phylogenetic relationships and inferring the origin of virus

**Fig 3:**
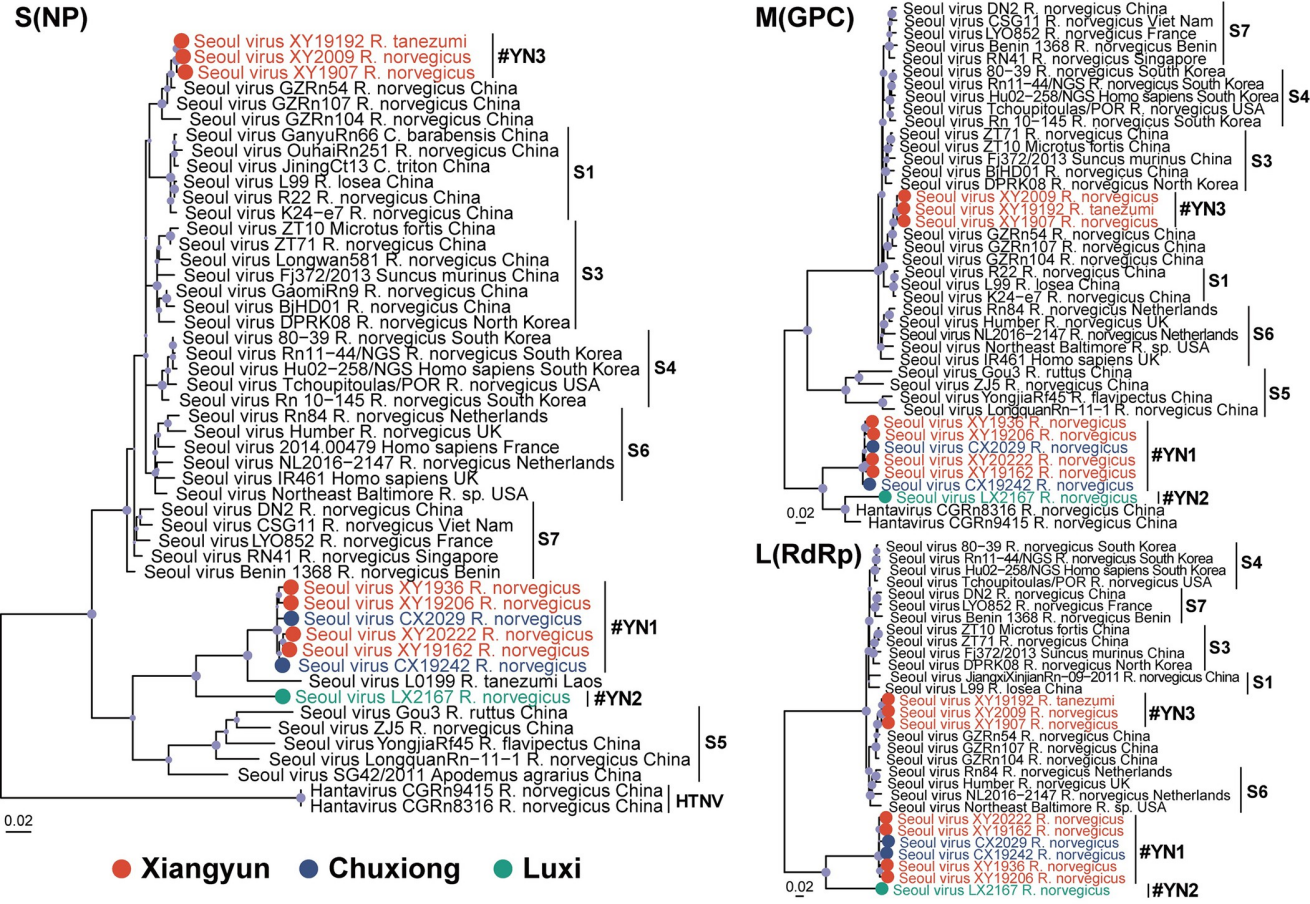
Phylogenetic trees based on the complete ORFs nucleotide sequences of S (1290 nt), M (3402 nt), and L (6456 nt) segment using the maximum likelihood method. Viruses detected in this study were represented by dots colored according to the geographical contribution.

Maximum likelihood phylogenetic trees reconstructed based on complete ORFs of the separate segments demonstrated a similar branching pattern and topology for L, M, and S genes (Fig 3), which were also consistent with the topology based on partial S segments (Fig 2A). We then compared these viruses with previous published sequences, YN3 shared close relationship with known virus strains GZRn54 identified from Guangzhou, China, whereas subgroup YN1 and YN2 were distantly related to existing strains. Indeed, they fell basal to other SEOV variants on the phylogenetic tree, in contrast. The same as homological analysis, the closest strain to YN1 was Seoul virus L0199 sampled from Laos, whereas the closest strain to YN2 was Hantavirus CGRn8316 sampled from Guizhou Province.

**Fig 4:**
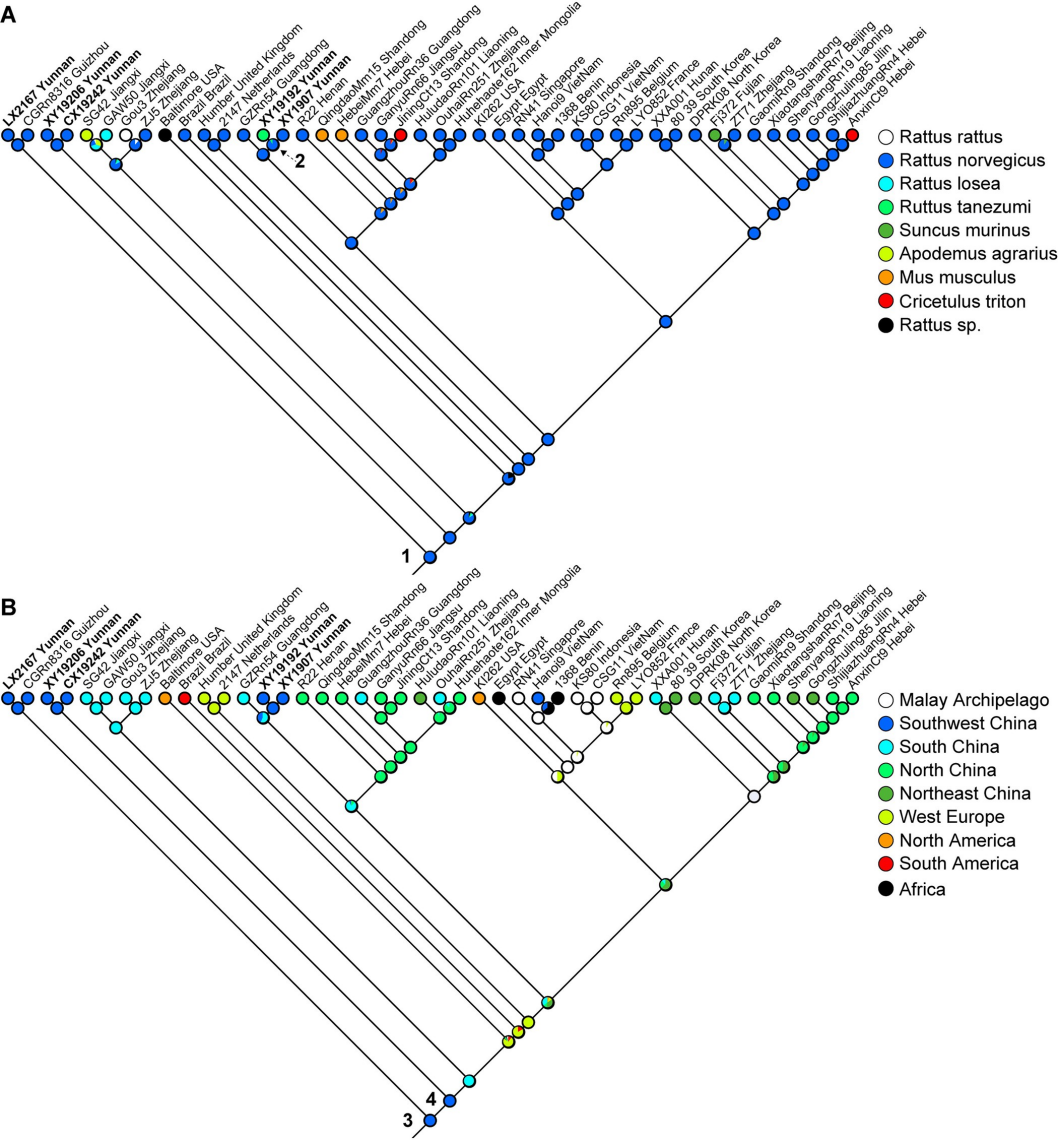
Phylogenetic distribution and ancestral character state reconstruction. Phylogenetic distribution and reconstruction of ancestral states for (A) the host species and (B) the biogeographic origin of SEOV using Mesquite 2.74 (with the uniquely best state for each node is displayed).

The ancestral character state reconstruction (ACSR) approach was performed to infer the ancestral hosts and biogeographic origin (ancestral range) of global SEOV. Partial M segment sequences of SEOV recovered from 9 mammal species in 14 countries of 5 continents were included in analyses. Above all, as shown in Fig 4A, the analysis reveals that the shared ancestral host (Node 1) of SEOV most likely be *Rattus norvegicus*, which was likely the main reservoir of SEOV. And the other carriers (such as *Rattus tanezumi*, Node 2) were most likely independent spillovers from the main reservoir. Subsequently, the viral sequences were re-grouped based on the geographic distribution into southwest China, south China, north China, and northeast China amongst others to infer the most likely ancestral ranges (Fig 4B). Since both of the basal lineages are associated southeast Asian and southwest China, the ancestral range for Node 3 and 4 were estimated to be the ancestral states, although an estimation of more precise locations of origin requires a broader sampling in Southeast Asia and Yunan province.

## Discussion

A total of 215 HFRS were reported in Yunnan province from 2005 to 2012 [42]; however, the disease was in a high incidence status since 2014, with 200 to 300 cases yearly [15]. The data revealed that 788 cases were documented from 2019 to 2021, suggesting that HFRS remains an increasingly serious public health issue in the province. Of these, 403 (51.1%) cases were from Xiangyun and Chuxiong (Fig 1D), suggesting these counties are still the main areas of HFRS endemicity in the province. Accordingly, we performed an investigation focusing on the causative hantaviruses (SEOV and HTNV) carried by rodents and shrews in these counties as well as a county (Luxi) where previous epidemic was recorded [16].

Epidemiological and clinical investigations have established SEOV as the primary causative agent of HFRS in Chuxiong and Xiangyun, with the viral variants YN1 and YN3 identified as capable of inducing HFRS [43–45]. Although previous study stated that HTNV was circulating in rodents in Luxi [14], however, no genetic or isolation evidence of HTNV was recorded before, and only SEOV was detected in the current study. Therefore, it remains to be seen whether exist co-circulation of the two viruses within a small region. The screening involving 1784 small mammals belonging to 14 species of three orders confirms that *Rattus norvegicus* is the predominant host of SEOV in the major endemic areas for HFRS of the province. The virus was also found harbored by *Rattus tanezumi*; however, the infection appears to be spillover transmission (host switch) from the predominate reservoirs (Node 2, Fig 4A). Notably, the HFRS cases in the endemic foci revealed the same trend with the prevalence of SEOV among reservoir rodents and the density of hosts in Xiangyun (Fig 1D). This strong correlation has been empirically tested and confirmed (S1 Fig). Therefore, monitoring and modeling rodent dynamics could be used to develop early warning systems for human epidemics [1]. However, the data available on HFRS dynamics and rodent surveillance in this study are limited, which could affect the reliability of the statistical tests. Additionally, the transmission dynamics of the disease are determined by complex and multifactorial mechanisms [30–40], which require continuous and multi-aspect surveillance.

SEOV was considered the only globally distributed hantaviral pathogen due to the omnipresence of its primary rodent reservoir [58]. The virus mainly circulates in Asia, with multiple isolations in four other continents (Europe, Africa, and both America) also documented [58, 59]. Although infected rats have been found in all provinces except Qinghai and Tibet, there was a predominance of SEOV in Southwest China [11, 60]. The phylogenetic and ACSR analyses reveal that the common ancestor of SEOV appeared to be originated near the southwestern areas (Yunnan-Kweichow Plateau) of China, then could spread east and north in China (Fig 4B). Coincidentally, some genetic studies conducted on modern specimens and the dated fossils that judged to represent ancestral forms of the Norway rats both implied that the species originated near the putative SEOV ancestral ranges (near Southwest China) [61,62]. The finding also confirms that the viral strains circulating outside China (such as in West Europe) seem directly originated from China (Fig 4B), which may follow the migration of their rodent reservoirs [63, 64]. Recently, the role of seaports as the source of hantaviruses has been recognized by the entry of hantavirus-infected rats [65–67]. Moreover, most of non-Chinese SEOV strains were found distributed along coastal lines [27,58], emphasizing the critical role of ship transports in spreading rodents and their carried pathogens. In summary, despite the accurate migration routes of SEOV spread remaining unclarified, it is clear that the virus originated from China and was later spread globally, most likely through its rodent reservoir. Nevertheless, the phylogenetic and biogeographic analyses are both highly dependent on the small and biased available virus sequences, restricting our understanding not only of virus evolution but also of geographic distribution, such that far more data are needed to clarify this issue.

The widely accepted co-evolution concept indicated that hantaviruses diverged from the single rodent-associated viruses millions of years ago, jumping to new hosts and adapting to the new environment drove the expansion of virus diversity [2,20,21]. The ancestral SEOV hosted by Norway rats appeared to diverge from the *Bandicota*-derived virus [10]. In the current study, three lineages of SEOV variant were discovered, including two that occupy the most basal position on the tree (Fig 3), furthering the understanding of the genetic diversity and ancient association of the virus. The molecular epidemical analysis suggests that the current distribution of different variants may be established by allopatric migrations and geographic separation of the viruses and their hosts. Whilst there is a co-circulation of two SEOV variants within a small region in Xiangyun (Fig 2B), the general congruence between the phylogenies of the different genome segments suggests no reassortment was observed (Fig 3). More areas in the province are now experiencing the prevalence of HFRS [15,42], implying the virus is spreading and diverse further. In summary, the recognition of the genetic diversity of viruses is influenced by numerous factors; however, it undoubtedly will be significantly expanding with the increasing sampling.

Overall, the growing data provided a more comprehensive understanding of SEOV regarding genetic diversity and ancestral ranges inference. At least three lineages of SEOV variant are circulating in rat populations in Yunnan. While recent findings have implicated the variants YN1 and YN3 in the etiology of HFRS [45], the absence of strain detected in patients in this study constitutes a limitation, and it remains unclear whether this diversity is associated with disease severity. Furthermore, few records of the virus were directly isolated/detected from humans in the province, warranting continuous surveillance focusing on HFRS patients.

## Supporting information

S1 Table Summary of animal hosts sampled from the main endemic areas of HFRS in Yunan Province from 2019 to 2021. (XLSX)

S2 Table Distribution of the reads for SEOV and their host COI gene of each library. (XLSX)

S3 Table Hantavirus strains and their GenBank information for those sequences used to perform phylogenetic analyses. (XLSX)

S4 Table Hantavirus strains and their GenBank information for those sequences used to perform ACSR analyses. (XLSX)

S1 Fig Correlations between HFRS cases, rodent density and SEOV prevalence in Chuxiong and Xiangyun from 2019 to 2020 determined by Spearman test. (TIF)
