# Supplementary figures and images for: The discovery of novel variants reveals the genetic diversity and potential origin of Seoul orthohantavirus
Source: PLoS Negl Trop Dis. 2024 Sep 12;18(9):e0012478. doi: 10.1371/journal.pntd.0012478 (PMC11392341; doi:10.1371/journal.pntd.0012478)

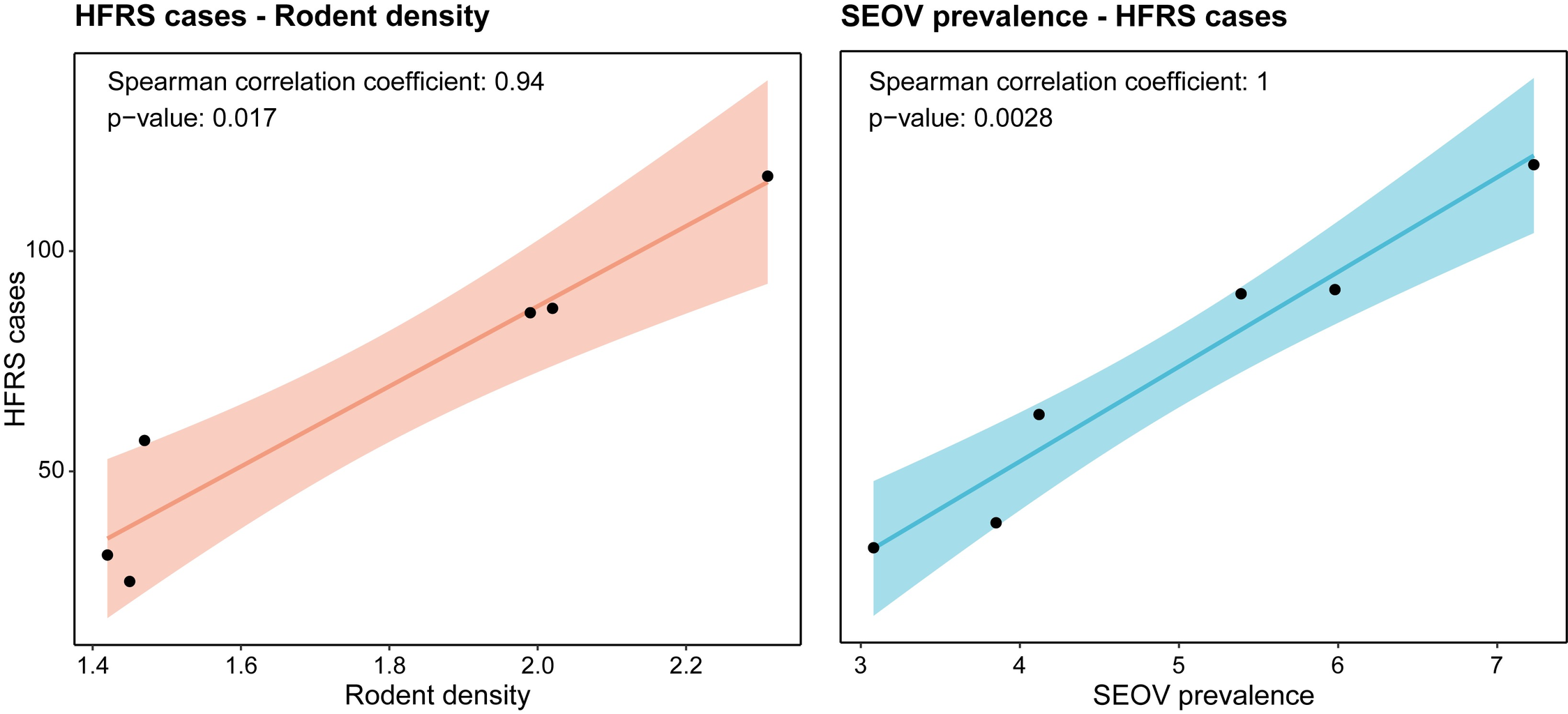

Supplement: S1 Fig — (TIF) [file pntd.0012478.s005.tif]
